# A Critical Review on Metal-Organic Frameworks and Their Composites as Advanced Materials for Adsorption and Photocatalytic Degradation of Emerging Organic Pollutants from Wastewater

**DOI:** 10.3390/polym12112648

**Published:** 2020-11-10

**Authors:** Zakariyya Uba Zango, Khairulazhar Jumbri, Nonni Soraya Sambudi, Anita Ramli, Noor Hana Hanif Abu Bakar, Bahruddin Saad, Muhammad Nur’ Hafiz Rozaini, Hamza Ahmad Isiyaka, Ahmad Hussaini Jagaba, Osamah Aldaghri, Abdelmoneim Sulieman

**Affiliations:** 1 Fundamental and Applied Sciences Department, Universiti Teknologi PETRONAS, Seri Iskandar 32610, Malaysia; anita.ramli@utp.edu.my (A.R.); bahruddin.saad@utp.edu.my (B.S.); Muhammad_18000735@utp.edu.my (M.N.H.R.); hamza_18001996@utp.edu.my (H.A.I.); 2 Chemistry Department, Al-Qalam University Katsina, Katsina 2137, Nigeria; 3 Chemical Engineering Department, Universiti Teknologi PETRONAS, Seri Iskandar 32610, Malaysia; soraya.sambudi@utp.edu.my; 4 School of Chemical Sciences, Universiti Sains Malaysia, Gelugor 11800, Malaysia; hana_hanif@usm.my; 5 Civil Engineering Department, Abubakar Tafawa Balewa University, Bauchi 740272, Nigeria; ahjagaba@atbu.edu.ng; 6 Physics Department, College of Science, Al-Imam Muhammad Ibn Saud Islamic University, Riyadh 11432, Saudi Arabia; odaghri@gmail.com; 7 Radiology and Medical Imaging Department, College of Applied Medical Sciences, Prince Sattam Bin Abduaziz University, Alkharj 11942, Saudi Arabia; a.sulieman@psau.edu.sa

**Keywords:** adsorption, emerging pollutants, metal-organic frameworks, photocatalytic degradation

## Abstract

Water-borne emerging pollutants are among the greatest concern of our modern society. Many of these pollutants are categorized as endocrine disruptors due to their environmental toxicities. They are harmful to humans, aquatic animals, and plants, to the larger extent, destroying the ecosystem. Thus, effective environmental remediations of these pollutants became necessary. Among the various remediation techniques, adsorption and photocatalytic degradation have been single out as the most promising. This review is devoted to the compilations and analysis of the role of metal-organic frameworks (MOFs) and their composites as potential materials for such applications. Emerging organic pollutants, like dyes, herbicides, pesticides, pharmaceutical products, phenols, polycyclic aromatic hydrocarbons, and perfluorinated alkyl substances, have been extensively studied. Important parameters that affect these processes, such as surface area, bandgap, percentage removal, equilibrium time, adsorption capacity, and recyclability, are documented. Finally, we paint the current scenario and challenges that need to be addressed for MOFs and their composites to be exploited for commercial applications.

## 1. Introduction

Emerging organic pollutants have received much concern due to their ubiquitous detection in various water spheres. They are toxic species produced from both natural and anthropogenic sources via; volcanoes, bush burning, petroleum exploration and refining, coal mining and processing, petrochemicals production, agrochemical application, textile, and leather dyeing, pharmaceutical production among others. They are widely discharged into the environment and conversely get deposited into the water bodies. Most of these pollutants are highly hydrophobic; thus, they bioaccumulate and magnify in the water and consequently get into the tissues of various aquatic organisms, as well as humans. Among the prominence includes dyes [1,2], pharmaceuticals and personal care products (PPCPs) [3,4], phenolics [5], herbicides and pesticides [6,7], polycyclic aromatic hydrocarbons (PAHs) [8,9], and perfluoroalkyl carboxylates and sulfonates [10,11]. These pollutants had been classified as endocrine disruptors (EDCs), due to their tendency to interfere with the function of the natural hormones [12,13]. They are highly resistant to naturally occurring processes of biodegradation and photolysis [14]. Toxicity studies have linked these compounds with many forms of ailments, such as genotoxicity, neurotoxicity, reproductive toxicity, development toxicity, cancerous tumors, etc. [15,16]. Thus, due to their frequent detection in the water and high toxicities, they are classified as emerging pollutants.

Environmental scientists, engineers, as well as environmental control and monitoring agencies, were challenged to provide effective remediations of these toxic pollutants. Thus, various methods have been put forward to achieve the tasks. Flocculation as an alternative have been practiced for decades [17]. The method is based on the formation of suspended solid particles (known as flocculants) using alumina, biopolymeric pectin, polyacrylamide, etc. [18]. Similarly, coagulation has also been considered [19]. However, the two suffered disadvantages of incomplete removal of the pollutants, as well as the formation of secondary pollution in form of sludge [20,21]. Other physical techniques, such as sedimentation, filtration, and reverse osmosis, have also been applied [22,23]. In most cases, they are not without drawbacks. Reverse osmosis, for example, requires periodic maintenance due to the clogging of the membranes [24,25]. The use of bioremediation using naturally occurring microorganisms, such as algae, bacteria, and fungi, to degrade the organic pollutants have been put forward [26,27]. However, some of these pollutants are resistant to biodegradations.

Due to the shortcomings of the aforementioned techniques, and driven by the need for a cheaper, sustainable, and effective treatment process, alternative approaches are necessary. Of these, adsorption and photocatalytic degradation are attractive as they could offer complete removal and mineralization of the toxic contaminants. This article is aimed at reviewing the application of metal-organic frameworks (MOFs) as versatile and highly efficient materials remediations of toxic organic pollutants from wastewater. Different classes of the pollutants have been discussed, and the literature reported on their removals by the MOFs has been detailed. Emphasis has been paid to adsorption and photocatalytic degradation using various pristine MOFs and their composites.

### 1.1. Adsorption

The application of adsorption techniques as an alternative wastewater remediation process has been well discovered. It has been proposed to solve the challenging task of incomplete removal of pollutants during wastewater processing. Organic pollutants are particularly more resistant to many forms of water remediation due to their hydrophobicity and lower molecular weight. For adsorption, process, pollutant molecules are attracted onto the surfaces of the adsorbent materials through diffusion process from the bulk of the solution to the active pores of the adsorbents [28,29]. Usually, the mechanism takes place through intermolecular forces of attraction, such as chemisorption (e.g., ionic interactions) and physisorption (e.g., van der Waals and π–π interactions) [30,31]. Adsorption has been emphasized by the unique properties of the adsorbent materials, such as high porosity, large specific Brunner Emmett Teller (BET) surface area, moisture and thermal stabilities, good selectivity for the target pollutants, availability, and low-cost, easy to handle and regenerated, etc. [32,33]. Among the desirable properties of ideal adsorbent materials is the physical state in form of either powder, cake or beads.

Among the most widely applied carbonaceous porous materials include biochar, activated carbon (AC), graphene, and carbon nanotubes [34,35]. They are usually obtained or synthesized from agricultural waste products. AC has been the most reported carbon-adsorbent. It has well-developed pore size distribution, with high surface functional groups that provide binding sites for adsorption of pollutants in water (surface area up to 1100 m^2^ g^−1^, and specific pore volumes up to 0.40 m^3^/g) [36]. Thus, it has found wide applications in water and gas purification, as well as separation processes [37]. Commercial AC is obtainable from non-renewable starting materials, such as lignite, coal, and petroleum coke. Although, there is a strong drive in using renewable materials, such as agricultural wastes (e.g., rice husks, fruit peels, sugarcane bagasse) [38,39]. AC, unfortunately, is not the ideal adsorbent material for treating emerging organic pollutants in water mainly due to the lack of complete removal at low concentrations. Furthermore, the time required for the adsorption is rather slow and the difficulty of regeneration of the used adsorbent. Progress in materials science has resulted in the introduction of new generation of adsorbents with abnormally high surface areas and porosity. These materials include mesoporous silica [40,41], halloysite nanotubes [42,43] graphene [44], molecularly imprinted polymers (MIPs) [45], and MOFs (e.g., MOF-5, HKUST-1, MIL-100, UiO-66, etc.) [46,47]. Significant selectivity can be achieved from the cavity size of the MOFs frameworks. Surface chemical modifications of these adsorbents usually brought about higher removal capacities and selectivity of the composites towards the organic pollutants.

### 1.2. Photocatalysis

Photocatalysis is a general term used to a defined catalytic reaction that is induced by light energy [48]. Of much interest is the potential of harnessing solar energy. It is an advanced oxidation process for the efficient degradation of toxic pollutants from wastewater using photocatalytic materials. In the process, the light energy is converted into chemical energy with the generation of free radicals, such as hydroxyl radicals, which attack the pollutants and subsequently degrade them into non-toxic by-products [49,50]. Thus, the field has attracted tremendous interest because of its advantageous features as summarized below:

(i) Ability to degrade pollutants within a short time with the help of light or solar energy.
(ii) Operates under ambient conditions.
(iii) Mineralization of organic pollutants into carbon dioxide and water; thus, no secondary pollutants are produced.

An ideal photocatalyst should be stable in both aqueous and organic solvents under acidic or alkaline solutions and be able to tolerate strong light irradiation. Additionally, it must be of high porosity, low-cost, have simplicity in applications, and be easily regenerated. Thus, various porous materials have been discovered. Among them, those containing mesopores and microspores have received much attention due to their uniformity in their surface morphology, particle size, pore volume, and diameters [51]. Some of these materials, such as MOFs, zeolites, silicates, graphene and reduced graphene oxide (GO and RGO), metal-oxide nanoparticles (MNPs), carbon quantum dots (CQDs), and other nanoporous carbon materials, can be chemically modified for the intended application. Of these, MOFs have shown lots of promise.

### 1.3. Metal-Organic Frameworks

**Figure 1:**
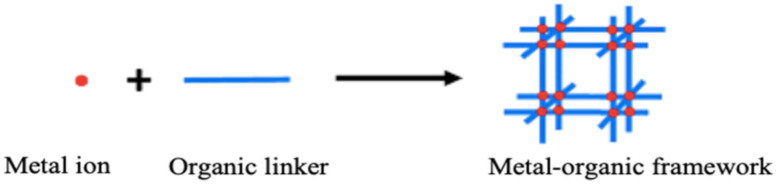
The schematic diagram for the formation of the metal-organic framework (MOF) from metal ion and organic linker as precursors. Reproduced with permission from Reference [57].

MOFs are advanced porous hybrid materials that are formed from coordination interactions of the metal node with organic linkers (Figure 1) forming two or three-dimensional structures of porous frameworks [52]. They are also referred to as a special group of Coordination polymers (CPs) involving strong metal-ligand interactions [53] and possessed metal-ligand coordinative bonds which are stronger than hydrogen bonds, and they have more directionality than other weak interactions, such as *π*-*π* stacking [54]. The development of porous materials can be traced back to 1990 from the work of Hoskin and Robson (1990) for the synthesis of scaffolding-like structural 3D frameworks by linking tetrahedral or octahedral arrays of metals centers with the organic moieties. A diamond-like framework, [N(CH_3_)_4_][CuZn(CN)_4_), having several cavities, was successfully synthesized and analyzed by single-crystal x-ray diffraction [55]. The group of Yaghi (1995) has been instrumental in the design new structures from the assembling of metal ions coordinated to the organic moieties as linkers. In 1999, the famous MOF-5 was successfully synthesized by the group [56], heralding the beginning of the exploration of novel structures of various dimensional frameworks.

Interest in MOFs is due to their peculiarities, uncommon to other synthetic materials, possessing ultra-high surface area, high crystallinity, uniformity of pore sizes, and tunability of volumes. Their microporous structures provide surface area of up to 9000 m^2^ g^−1^ and specific pore volumes of up to 2 cm^3^ g^−1^, together with a large variety of pore dimensions and topologies. The unique features of MOFs found numerous applications in gas storage, CO_2_ capture and conversions, chemical separations, drug delivery, nerve agents, sensing, energy conversion, pre-concentrators of explosive vapor, catalysis, wastewater remediations, etc. [58,59].

MOFs possessed open-framework structures that can allow for the inclusion of guest species, particularly solvents during synthesis. These guest species could be removed via desolvation that may result in an empty framework [60]. Therefore, the nature of the framework is determined by the extent to which the volatile solvents are sufficiently removed or exchanged to permit either the generation of a truly porous material or other molecules to occupy the pore structure [61,62]. The MOFs system allows access to open-framework structures with network topologies and connectivity that are not usually observed in classical porous materials [63]. Of much interest is the possibility of generating large-diameter channels and cavities. By controlling the size and functionalization of the organic linkers, well defined MOF structures with high surface areas and tunable pore sizes can be achieved [64,65].

Few reviews were found in the literature highlighting the applications of MOFs for wastewater remediation. Kumar et al. (2018) focused on inorganic contaminants removal using MOFs in the wastewater system [66]. A review by Dhaka et al. (2019) also discussed more on the performance of MOFs for the adsorptive removal of several emerging pollutants [67]. In addition, the performance of MOFs on heavy metals and other inorganic pollutants removal compared to other adsorbents. Joseph et al. (2019) also reviewed the removal of pharmaceuticals drugs in wastewater [68]. However, those reviews have not discussed details on adsorption of various classes of emerging organic and that the photocatalytic degradation of the pollutants was not considered. The present review is aimed at filling the gaps that were not provided by the earlier reports. Thus, a comprehensive update on the adsorptive removal of emerging organic pollutants, using MOFs and their composites are presented. Additionally, the photocatalytic degradation of these pollutants by the MOFs and composites will be discussed. Since the effectiveness of an adsorbent is normally evaluated based on adsorption capacity, selectivity for the specific compound, and regenerability, these relevant data and others are provided in our compilations.

## 2. MOFs for Remediation of Emerging Pollutants in Water

### 2.1. MOFs for Adsorption

The possibility to synthesize hundreds of frameworks from various clusters of metal ions with organic linkers gives rise to an unlimited number of crystalline MOFs with microporous or mesoporous structures. Additionally, different functional groups in the organic linkers and metal node serves as adsorption centers for various types of organic contaminants [69].

**Figure 2:**
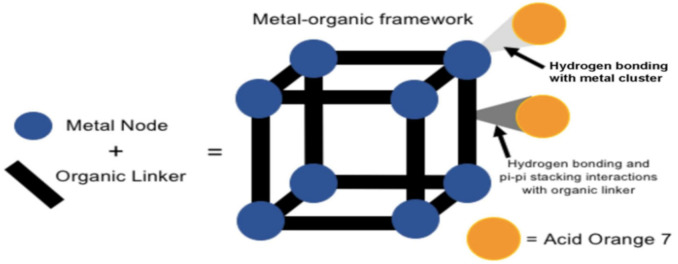
Interactions in adsorption of a contaminant (acid orange 7) onto the pores of MOFs. Reproduced with permission from Reference [76].

MOFs also offer selective adsorption of organic molecules due to the functionalities of the organic linkers, possibly forming inclusion complexes with the guest adsorbate molecules. The mode of adsorption interactions is usually through covalent bonding, hydrogen bonding, dative bonding, Van der Waals forces, and π-π interactions [70,71] (Figure 2). Molecular modeling has shown that when the pore sizes of the MOF is bigger than the pollutant molecule, the guest molecule to preferably resides in the pores of MOFs [72]. Alternatively, the guest molecule is adsorbed on the outside if it is bigger than the pores of the MOF. Thus, choosing the MOF for the adsorption of an analyte is important to optimize the adsorption [73,74]. MOFs with promising adsorption properties have been selectively used for the removal of contaminants in water. Their stabilities, adsorption capacities, and ease of reusability have been reported [75].

**Figure 3:**
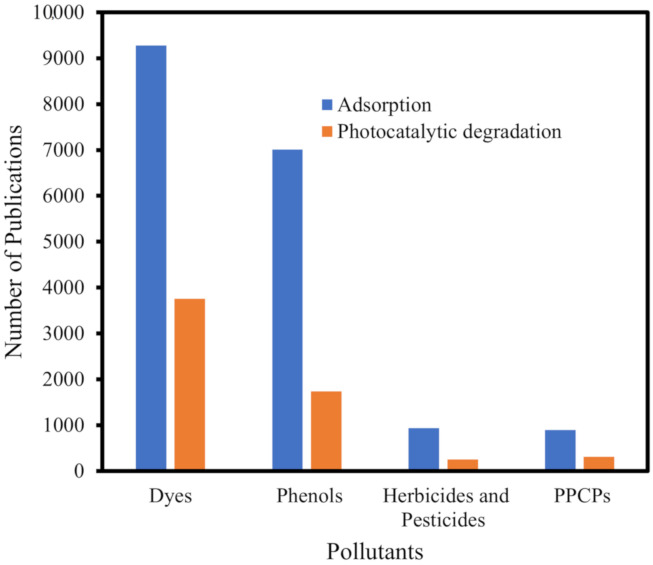
Publications on the adsorption and photocatalytic degradation of some emerging pollutants using MOFs from 2010–2020. Data were obtained from science direct using the keywords; MOFs; adsorption; photocatalytic degradations; dyes, phenols; pesticides and herbicides; and pharmaceuticals and personal care products PPCPs.

For the past 10 years, MOFs have received considerable attention as potential adsorbent materials for the removal of pollutants in water. The number of articles that were published from 2010–2020 on the adsorption and photocatalytic degradation by MOFs according to the category of pollutants is shown in Figure 3. It can be readily seen that publications were predominantly on adsorptions compared to photocatalytic degradation. Dyes were also popular topics of research both for adsorption and photocatalytic degradation. This is not surprising as studies on removal and degradation of dyes are easy to be executed using spectrophotometers, and the effects can be seen with the naked eye. On the other hand, studies on pollutants that are not chromogenic, such as the Perfluorooctane sulfonates (PFOS) and Perfluoroalkyl substances (PFAS), will require less readily available instruments, such as High performance liquid chromatography (HPLC)-conductivity or tandem HPLC-MS. Nevertheless, it can be expected that studies using MOFs for other categories of pollutants will grow significantly in the coming years.

### 2.2. MOFs for Photocatalysis

The idea of using MOFs as photocatalysts were first conceived by Alvaro et al., 2007 [77], when investigating the semiconducting properties of MOF-5. In their pioneering studies, terephthalate organic linker of the MOF, when in solution tends to generate some changes. This is suggested by the fact that electrons are ejected from the excited terephthalate molecule. This finding was the catalyst for investigations on the use of MOFs as photocatalyst for the degradation of different contaminants in water.

Generally, MOFs exhibit semiconductor-like behavior upon light irradiation. The organic linker can act as an antenna to harvest light from the either natural or artificial sources and subsequently activate the metal sites via ligand to metal cluster charge transition (LMCT) [78]. The mechanism can be viewed in terms of excitation of an electron from the highest occupied molecular orbital (HOMO) to the lowest unoccupied molecular orbital (LUMO) when light is irradiated on the MOF, thus leaving a hole in the HOMO. This hole can interact with OH, forming an OH^•^ radical which oxidizes the organic compounds [79]. Thus, the photocatalytic performance of the photoactive MOF involves the generation of electron-hole pairs in the conduction and valence bands of the MOF respectively. In an aqueous medium, the generated electrons (e^−^) interact with oxygen to produce oxygen radicals which in turn transform to hydroxyl free radicals (OH^•^). Similarly, the generated holes (h^+^) could undergo a reduction upon interactions with the hydroxyl molecules in the solution to form the hydroxyl free radical or act directly or the pollutant. In both cases, the OH^•^ and h^+^ active species could sufficiently attack the target pollutant and subsequently breaks all the bonds in the analyte to ultimately form non-toxic species (CO_2_ and H_2_O). Thus, an important criterion in the choice of MOFs for photocatalytic applications is the ability of the MOFs to harvest and channel the light energy.

The high porosity of MOFs contributes extensively to the photocatalytic process by trapping the pollutants. Some MOFs containing Fe, Cr, Zr, and Ti metal ions exhibit good stability in water and can harvest and channel solar energy [80]. They usually possess a small bandgap which enables visible light excitation; hence, they are considered as good candidates for photocatalytic degradations of organic pollutants [81].

### 2.3. MOF Composites for Adsorption and Photocatalytic Degradation

Even though the fact that MOFs have displayed good potential as adsorbents and photocatalysts for pollutant remediation, some MOFs are plagued by poor chemical and moisture stability and the inability to harness energy from sunlight. To overcome these shortcomings, MOFs have been incorporated with other functional materials, such as metal and metal-oxide nanoparticles (MIL-101(Cr/Al)) [82,83], carbon quantum dots (CQDs/NH_2_-MIL-125(Ti)) [84], graphene and graphene oxides, zeolite (ZIF-67@MIL-125-NH_2_) [85], (CNT@MIL-68(Al) [86], molecular imprinted materials e.g., polydopamine (PDA/Fe-MOF/RGO) [87], and ionic liquids, to form composites. These MOF composites were prepared using techniques, such as fabrication, impregnation, surface functionalization, immobilization, and deposition. Some of the methods were able to produce composite MOFs with remarkable properties than the precursor materials. Nevertheless, applications of composites of MOF as photocatalysts are still at the infancy stage. An important target of photocatalytic activities is low bandgaps (<3.0 eV) that allow visible light from the sun to be harnessed.

The MOFs composites usually possessed some synergistic effects, such as the reduction of bandgap, lower photoluminescence, and photocurrent response, to harness light energy and prevent electron-hole recombination. Thus, the composites are highly efficient in utilizing light energy from both visible and ultra-violet regions and higher stability in harsh environments as compared to the counterpart pristine MOFs [88,89].

Similarly, MOF composites with other active materials, such as metal-oxide nanoparticles, carbon quantum dots (CQDs), and graphene oxides (GO), have proven to be effective photocatalysts for the degradation of organic pollutants. This is because the incorporated semi-conductor materials help to facilitate electron transfer in the MOF, resulting into effective separation of the photogenerated electron-hole pairs. On this basis, Wang et al. (2019) [90] proposed on the mechanism of enhanced photocatalytic degradation of rhodamine blue dye using CQDs supported on NH_2_-MIL-125(Ti) as follows:

CQDs/NH_2_-MIL-125(Ti) + hv → CQDs/NH2-MIL-125 + e^−^ + h^+^ (1)

e^−^ + O_2_ → O_2_^•−^ (2)

O_2_ +H_2_O → HO_2_^•^ + OH^•^ (3)

HO_2_^•^ + H_2_O → H_2_O_2_ + OH^•^ (4)

H_2_O_2_ → 2OH^•^ (5)

h^+^ +OH^−^ → OH^•^ (6)

OH^•^ + RhB(dye) → CO_2_ + H_2_O (7)

h^+^ + RhB(dye) → CO_2_ + H_2_O (8)

**Figure 4:**
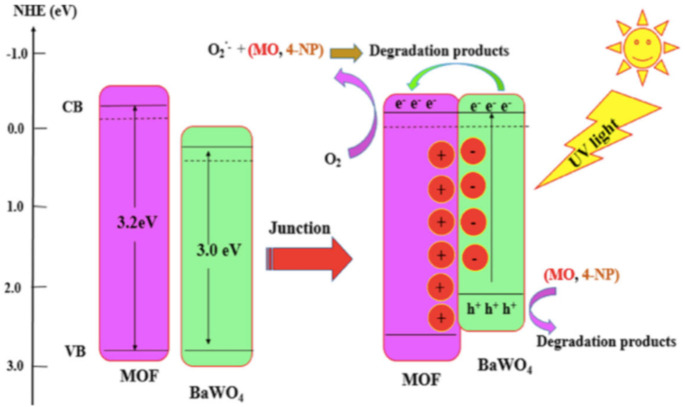
The mechanism for photocatalytic degradation of methyl orange and 4-nitrophenol using composite photocatalyst (MOF-199-NH_2_/BaWO_4_). Reproduced with permission from Reference [93].

Recently, Li et al. (2019) prepared an interesting heterojunction composite of the MOF (NH_2_-MIL-53(Fe)) with graphitic carbon nitride doped pyromellitic to form the composite (g-C_3_N_4_/PDI@NH_2_-MIL-53(Fe)) using the facile hydrothermal technique. The composite exhibited photoactive for the removal of tetracycline (90% in 1 h), carbamazepine (78% in 2.5 h), bisphenol A (100% in 10 min), and p-nitrophenol (100% in 30 min). Additionally, the composite MOF was more efficient in terms of reusability (5th cycles for each pollutant) than the pristine NH_2_-MIL-53(Fe) MOF [91]. Similarly, the synthesis of hybrid MOF/COF composites of NH_2_- MIL-53(Al), NH_2_-MIL-125(Ti), and NH_2_-UiO-66(Zr) with (1,3,5-triazine-2,4,6-triyl)tribenzaldehyde (TTB) and 4,4′,4″-(1,3,5-triazine-2,4,6-triyl)trianiline(TTA) to form N/TTB-TTA (N = NH_2_-MIL-53(Al), NH_2_-MIL-125(Ti), and NH_2_-UiO- 66(Zr)) was reported by the group of He et al. (2019). These hybrids MOFs have shown improved porosity and photocatalytic efficiency for the complete mineralization of methyl orange in aqueous medium [92]. Figure 4 depicted the mechanism of methyl orange and 4-nitrophenol degradation using MOF-199-NH_2_/BaWO_4_ composite synthesized from MOF-199-NH_2_ and BaWO_4_ by the immobilization technique [93]. It is interesting to note that the immobilization of BaWO_4_ into the MOF-199-NH_2_ has caused a red-shift in the absorption maximum of the composites with lower optical property than the pristine MOF. In addition, the calculated bandgap of the composite is lower (3.0 eV) compared to the MOF-199-NH_2_ (3.2 eV) (Figure 4). Thus, complete degradation within 50 and 80 min were achieved using the MOF-199-NH_2_/BaWO_4_ composite for methyl orange and 4-nitrophenol, respectively.

## 3. MOFs and Composites for Adsorption and Photocatalytic Degradation of Emerging Pollutants in Water

### 3.1. MOFs and Composites for Adsorption and Photocatalytic Degradation of Dyes

**Figure 5:**
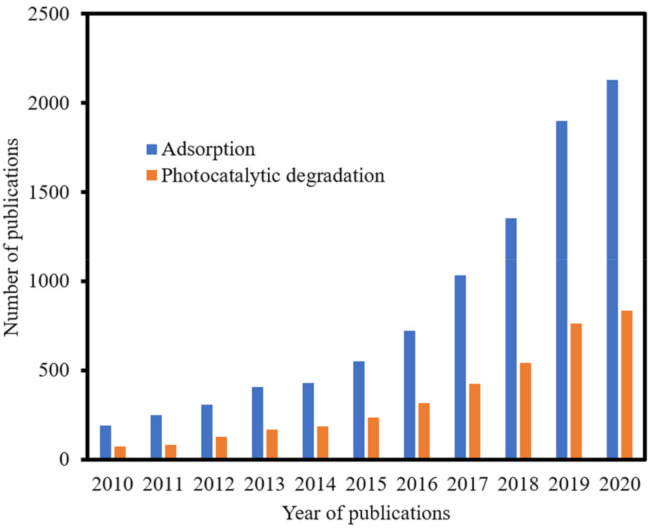
Publications from 2010–2020 on the adsorption and photocatalytic degradation of dyes using MOFs. Data was obtained from the science direct using keywords MOFs; adsorption, and photocatalytic degradations dyes.

Globally, water contamination from dyes has been one of the biggest sources of environmental pollution. Despite various regulations on the use of dyes, the discharge of effluents containing dyes, particularly from small-scale textile, cosmetics, leather, and food industries, has been a major source of water pollution. These dyes, when discharge into the environmental water, usually cause significant ecological threats, such as destruction of aquatic life, impeding plant growth, and posing various forms of toxicity to humans, including genotoxicity, reproductive toxicity, neurotoxicity, and other forms of diseases [21]. Thus, concerted efforts are needed to address the problem at the source and to remediate the already polluted water to safe levels. Figure 5 depicted the trends in publications on adsorption and photocatalytic degradations of dyes for the last decade. Exponential growth in the number of publications has been observed each year for both adsorptions and photocatalytic degradations. For instance, in 2020 alone, 2131 and 834 the number of articles has been reported on the adsorption and photocatalytic degradations of dyes, respectively, according to the data obtained from science direct repository.

**Figure 6:**
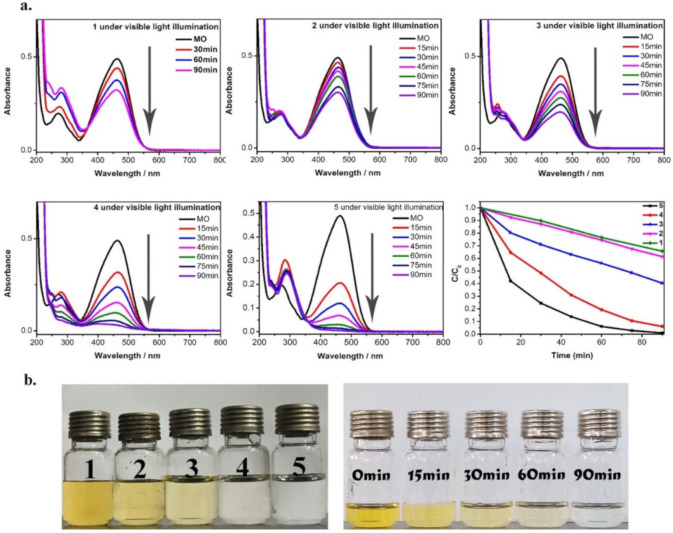
(**a**) Adsorption and photocatalytic degradations spectra of methyl orange dye using Zn and Co^2+^/Zn^2+^ metal-doped MOFs (M(tpbpc)(bdc)0.5·H_2_O) and (**b**) photographs of photocatalytic degradation of the dye using the MOFs under visible light irradiations. Reproduced with permission from Reference [97].

The significant porosity of MOFs due to the number of empty spaces within the frameworks rendered them a suitable candidate for dye adsorption [94]. The MOFs can provide larger adsorption sites for various kinds of dye molecules, including both cationic and anionic [95,96]. The simultaneous adsorption and photocatalytic degradation of methyl orange (Figure 6) using Co- and Zn-based MOFs, (M(tpbpc)(bdc)0.5·H_2_O) was reported by Liu et al. (2017), with complete mineralization of the dye achieved at 90 min of irradiations [97].

Table 1 summarizes some of the properties of MOFs as adsorbents for the removal of dyes from water. Some of the MOFs exhibited abnormally high surface area (up to 3500 m^2^ g^−1^). More so, they have shown higher adsorption capacities than other conventional adsorbents. For example, UiO-67(Zr) was able to achieve an equilibrium adsorption capacity of 799 mg g^−1^, for Congo red adsorption [98]. Adsorption capacity with (q_e_) value of 1045 mg g^−1^ was achieved for the adsorption of methylene blue by MIL-100(Fe) [99]. It is heartening to note that some of the MOFs were able to achieve almost or complete removal of the dyes within a relatively shorter time than the other adsorbents, which take several days to achieve complete removal. Many authors did not report the regeneration of their adsorbents; nevertheless, some of these MOFs could be reused a number of times without significant reduction in their efficiencies.

With the discovery of the photocatalytic properties of the MOF-5 in 2007, researchers continue exploring the photocatalytic efficiencies of other classes of MOFs for the degradations of contaminants from wastewater, of which dyes received considerable attention. The photocatalytic degradation offers an interesting option to completely breakdown the persistent dyes into neutral species. Some of the MOFs reported were able to degrade the contaminants under sunlight irradiations due to their lower band-gap, higher surface area, and pore volume, as well as good stability in aqueous medium. However, a major shortcoming encountered was the inability of some MOFs to be activated under visible light irradiations. Similarly, some of the MOFs were unstable in an aqueous medium. As such modifications using functionalized materials were considered [128]. Thus, various MOF composites, such as bi-metallic MOFs [129], NPs@MOFs [130], CQDs@MOFs, etc., with different active species were found to be more effective than the corresponding pristine MOFs, particularly in terms of harvesting visible light, preventing electron-hole recombination, and reusability.

Some MOFs and their composites reported for the photocatalytic degradation of dyes are summarized in Table 2. It can be seen that some of the pristine MOFs possessed high bandgaps (>3.0 eV); thus, they cannot utilize visible light effectively for photodegradation to occur, particularly under the sunlight irradiations. However, it is worthy to note that, the higher surface area of the MOFs might result in their higher absorption profile which can be extended to the visible region. Thus, they can absorb a few photons of visible light energy, capable to generate some holes on the surface of the MOFs to form free radicals that can act on the dyes. The functionalization of the organic linker in the MOFs was also responsible for the photocatalytic degradation. As an example, the presence of NH2 in NH2-MIL-88(Fe) has been claimed as the contributing factor to the adsorption capacity due to the shift in the absorption maximum of the MOF [50]. It is interesting to note that, modifications of the MOFs with light active species, such as metals, metal oxides, sulfides, etc., resulted in MOF composites with much lower bandgaps than the pristine MOFs or the active materials themselves [131]. Of all the MOFs reported in Table 2, only 16% were able to achieve the bandgap of less than 3.0 eV. This underscores the need for new materials with reduced bandgaps to tap sunlight irradiation for their degradation.

### 3.2. MOFs and Composites for Adsorptive Removal and Photocatalytic Degradation of Phenols and Other Miscellaneous Emerging Pollutants

Phenolic compounds are widely used by chemical and allied industries in making useful products, such as petrochemicals and plastics. Phenols and its derivatives are also used as a precursor in chemical industries in the production of pharmaceuticals, dyes, herbicides, pesticides, detergents, epoxies, among others. It has been estimated that more than 10 million tons of phenolic compounds are discharged annually into the environment, thus polluting the soil, surface water, and underground water [164]. The presence of these toxic endocrine-disrupting compounds, such as phenol, bisphenol A, 2,4-dinitrophenol, and 2,3,4,5-tetrachlorophenol, in the wastewater poses negative effects to living organisms, threatening the harmony of ecosystems [165]. The United States Environmental Protection Agency (USEPA) stipulates the threshold level of phenolic effluents to be discharged into public sewage systems should not exceed 5 ppm, and the maximum permissible limit in potable drinking water should not exceed 1 ppb [166].

Modern agricultural practice requires the use of agrochemicals, such as pesticides and herbicides, that help to protect farm products from pests, controlling unwanted weeds, as well as boosting the yield of crops. Herbicides are chemicals that are primarily produced to inhibit weeds that compete with the plant’s growth, while insecticides are aimed at repelling or mitigating insects and other pests from attacking the agricultural products, such as fruits, vegetables, cotton, etc. Commonly used agrochemicals are the neonicotinoids (e.g., thiamethoxam, imidacloprid, acetamiprid, nitenpyram, dinotefuran, clothianidin, and thiacloprid), organophosphates (e.g., diazinon, parathion, methyl parathion, paraoxon, and fenitrothion) and carbamates (e.g., aldicarb, carbaryl, and methomyl). When applied, these chemicals accumulate in the soil and subsequently washed into the environmental waters, such as lakes, lagoon river, and groundwater, posing potential hazards to the ecosystem [167]. Glyphosate, the most widely used herbicide in the USA, has been listed as a likely human carcinogenic agrochemical by the World Health Organization [168]. Similarly, atrazine also has been reported to show endocrine-disrupting property to aquatic animals even at low concentrations [169].

Other emerging pollutants of high toxic effects in water are the polycyclic aromatic hydrocarbons (PAHs). They are a group of hydrophobic compounds with two or more benzene rings. PAHs are known to originate extensively from anthropogenic sources, particularly from crude oil exploration, petrochemical effluents, oil spillage, etc. [170,171]. Due to their lipophilic nature, they are prone to be accumulated in the fatty tissues of living organisms. Long-term exposure to PAHs results in eye irritations, nausea, vomiting, and, in severe cases, may lead to liver and kidney failure and lung cancer [172,173]. Hence, they are categorized as emerging contaminants by the European Union, the USEPA, and other environmental regulatory bodies [174].

Another group of highly recalcitrant emerging pollutants that have currently gained world-wide attention are the poly and perfluorinated alkyl substances (PFAS). PFAS made headlines because they were found in the drinking water across many cities in the US and other countries of the world. Removing them is so difficult that scientists have nicknamed them “forever chemicals.”

**Figure 7:**
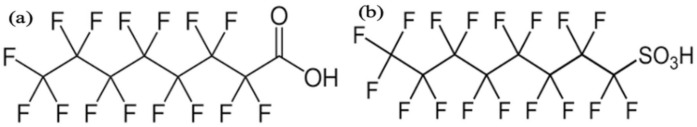
Molecular structures of (**a**) Perfluorooctanoic acid (PFOA) and (**b**) Perfluorooctane sulfonates (PFOS).

PFAS are fluorinated chemicals that have been widely used for the production of industrial (e.g., surfactants) and consumer products (e.g., non-stick coatings). The most toxic of these groups are the perfluoroalkyl carboxylates (PFCAs) and perfluoroalkyl sulfonates (PFAS). Perfluorooctanoic acid (PFOA) tends to bioaccumulate in human tissues and possessed a half-life of 4 years [175]. PFOA and PFOS are highly water-soluble; thus, they are readily transported in the aquatic environment. These compounds are detected in surface water [176], groundwater [177], rainwater [178], wastewater [179], and drinking water [180]. They have also been detected in a number of food matrices [181], human serum, breast milk, and other biological samples [182]. The USEPA has recommended clean-up of underground water that is contaminated with 70 parts per trillion of PFOA and PFOS [183]. The recommendation, however, is applied to groundwater that is a current or potential source of drinking water. The structures of PFOA and PFOS are shown in Figure 7. Typical of perfluoro compounds, it is the high-energy C–F bonds that render them persistent in the environment.

The toxicological impacts of these emerging pollutants have motivated researchers to look for green and environmentally sustainable methods for their remediations. Some water-insoluble MOFs and their composites offer good removal and photo-active degradations of herbicides and pesticides from wastewater. As an example, rapid (20–60 min) and complete removal (99%) of glyphosate were achieved using the highly porous zirconium MOFs NU-100(Zr) and UiO-67(Zr) [184]. Similarly, high removal of bisphenol A (473 mg/g) was achieved in 30 min using MIL-53(Al)-F127 composite MOF [185].

Studies by Apkinar et al. [186] exemplify the synthetic tunability of MOFs on the role of chemical functionality in the adsorptive removal of pollutants from water. The team investigated the adsorption in several Zr-based MOFs with a variety of pore sizes and with increasingly large conjugated π- systems and framework topologies. The unusually fast equilibration adsorption of 1 min exhibited by NU-1000 is due to the rapid diffusion through the hierarchically porous MOF structure although its capacity is comparable to that of other adsorbents that have been used for atrazine adsorption. The studies further corroborated that the presence of linkers with extended π-systems, rather than large pores results in the exceptional atrazine uptake by NU-1000. The applications of some of the MOFs and their composites as adsorbents and photocatalysts for the remediations of these water pollutants are summarized in Table 3 and Table 4.

**Figure 8:**
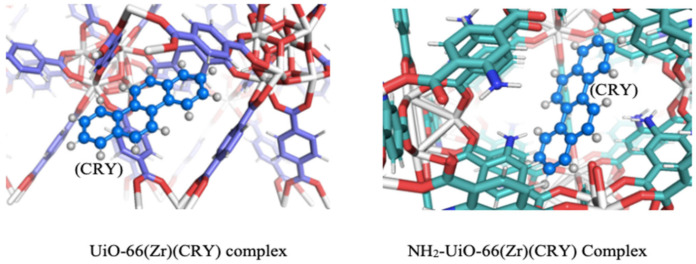
Diagram for the molecular docking simulation for adsorption of chrysene onto UiO-66(Zr) and NH_2_-UiO-66(Zr) MOFs (showing the pollutant in the inner pores of the UiO-66(Zr) and the outer pores of the NH_2_-UiO-66(Zr)). Reproduced with permission from Reference [187].

Recently, we reported the adsorptions of PAHs in aqueous medium using the highly porous Zr-based UiO (UiO-66(Zr), NH_2_-UiO-66(Zr)) [187], and MILs (MIL-88(Fe) and NH_2_-MIL-88(Fe) [188,189]. In most cases, rapid adsorption of the pollutants was achieved within a short time (30 min), which were attributed to the availability of the active adsorption sites in the MOFs. Molecular docking simulation was used to study the fundamental interactions between the MOFs with chrysene as a PAH model compound (Figure 8). The binding interaction studies show that the chrysene preferably resides in the inner and outer pores UiO-66(Zr) and NH_2_-UiO-66(Zr), respectively. The preference has resulted from the pore diameters of the MOFs concerning the molecular size of the pollutant [187].

Very limited reports can be found on the use of MOFs for the adsorption of the perfluoro compounds (Table 3). Jun et al. (2019) investigated the competitive adsorption of three adsorbates (i.e., bisphenol A, 17α-ethynyl estradiol, and PFOA) using Al-MOF. The effects of various water chemistry parameters, such as solution temperature, pH, background ions, and natural organic matter (i.e., humic acid), were also studied. The authors concluded that the synergetic effects of hydrophobic and electrostatic interactions were important factors in the adsorption process. Three MOFs, zeolitic imidazolate framework-7 (ZIF-7), ZIF-8, and ZIF-L were investigated for the adsorption of PFOA in an aqueous solution by Chen et al. (2016). The PFOA sorption performance of ZIF-7, ZIF-8, and ZIF-L was then compared with the performance of two commercialized adsorbents, zeolite 13X and activated carbon. ZIF-8 and ZIF-L were shown to outperform the two commercial sorbents. Their work demonstrates that the crystal structure and the surface functionality of MOFs influence, PFOA adsorption performance. To date, there is yet to be found reports on the photocatalytic degradation of perfluoro compounds using MOFs and composites.

Some articles published for photocatalytic degradations of phenols, pesticides, herbicides, and PAHs using MOFs and their composites are found in Table 4. According to a report by Mei et al., 2019, complete mineralization of thiamethoxam was achieved within 60 min of visible light irradiation in the presence of MIL-53(Fe) [190]. Before that, Ahmad et al. (2018) decorated MIL-100(Fe) with for ZnO nanosphere for the degradation of phenol, bisphenol A and atrazine. The introduction of the ZnO into the MOF has boosted its optical property; hence the composite was able to absorb visible light. More than 90% of the pollutants were degraded within 120 min [191]. Recently, photocatalytic degradation of bisphenol A was reported using MOF@COF hybrid composites of Fe-MIL-101-NH_2_@TPMA and Zr-UiO-66-NH_2_@TPMA. The synergetic effect of the persulfate (PS) added to the medium coupled with the optical properties of the composites was able to degrade 99% of the pollutant within 240 min under visible light irradiation [192]. To date, MOF has not been reported for the photocatalytic degradations of PAHs and PFASs. A difficulty in the detection of PFASs has been considered as a challenging factor, as it requires sophisticated tandem mass spectrometry.

**Table 3:** MOFs and composites used for the adsorptions of phenols, herbicides, pesticides, and other miscellaneous organic pollutants.

Type of MOF	Synthesis Method	Surface Area (m^2^ g^−1^)	Pollutants	Concentration (mg L^−1^)	% Removal	Qe (mg g^−1^)	Equilibrium Time	Reused	Ref
			**Phenolics**						
MIL-53(Al) MIL-53(Al)-F127	Hydrothermal	931 1008	Bisphenol A	250	-	329 473	90 min 30 min	3 3	[185]
MIL-68(Al)/PVDF	Casting	-	P-nitrophenol	10	94	126	720 min	6	[125]
HKUST-1(Cu)	Microwave	-	P-nitrophenol	200		400	30 min	-	[193]
SiO_2_@MIL-68(Al)	Solvothermal	1156	Aniline	3000	-	532	40 s	5	[194]
[Zn(ATA)(BPD)] MOF-VII	Ultrasound	170 675	2,4-dichlorophenol	60	68 91	- -	90 min 90 min	5	[195]
[Zn(TDC) MOF	Vapor-diffusion	235	2, 4-dichloropheno	60	95	-	180 min	-	[196]
MIL-68(Al) CNT@MIL-68(Al)	Solvothermal	1283 1407	Phenol Phenol	1000	- -	118 257	120 min	5	[86]
NH_2_-UiO-66(Zr)	Solvothermal		2,4,6-trinitrophenol Styphnic acid 2,4-dinitrotoluene	100	-	23 24 0.5 2	36 h	-	[197]
MIL–68(Al) MIL–68(Al)/GO	Solvothermal	550 762	p–nitrophenol	300	- -	271 332	17 h 17 h	5	[198]
NH_2_-MIL-88(Fe)	Hydrothermal	414	2,4,6-trinitrophenol	35	-	164	40 min	5	[199]
MOF-199(Cu)	Solvothermal	2271	Phenol *p*-nitro phenol	500	80 89	58 68	300 min 30 min	- -	[200]
Al-MOF/SA-CS	Hydrothermal	688	Bisphenol A	50	-	137	18 h	6	[201]
Cu-BDC MOF Cu-BDC@GrO Cu-BDC@CNT	Solvothermal	- - -	Bisphenol A Bisphenol A Bisphenol A	200	97	60 182 164	40 min	5	[202]
laccase@HKUST-1	Immobilization	-	Bisphenol A	200	74	-	4 h	NA	[203]
			**Pesticides**						
M-MOF	Room temperature	250	Thiamethoxam Acetamiprid Nitenpyram Dinotefuran Clothianidin Thiacloprid	100	-	3 3 3 3 2 3	60 min	-	[204]
MIL-101(Cr)	Hydrothermal	2612	Diazinon	50	54	158	45 min	4	[205]
Cr-MIL-101-BTP	Hydrothermal	1113	Acetochlor	120	100	322	200 min	6	[206]
MIL-101(Cr) TS-MIL-101(Cr)	Hydrothermal	-	Atrazine	30	37 69	-	60 min	-	[207]
			**Herbicides**						
HKUST-1(Cu) ZrO_2_@HKUST-1	Room temperature	1484 1152	Cyhalothrin	60	-	140 138	2 h	-	[208]
UiO-67(Zr)	Hydrothermal	2172	Glyphosate Glufosinate	200	96 92	537 360	150 min 200 min	-	[209]
NU-100(Zr) UiO-67(Zr)	Solvothermal	N/A N/A	Glyphosate	1117.5	100 100	1340 1500	20 min 60 min	- -	[184]
UiO-66(Zr) UiO-67(Zr)	Solvothermal	1640 2345	Atrazine	25	20 98	3 12	50 min 2 min	1 4	[210]
DUT-52(Zr) NU-1008(Zr) NU-901(Zr) NU-1000(Zr)	Solvothermal	1960 1400 2110 2110	Atrazine	10	82 69 85 93	-	1 min	3	[186]
			**PAHs**						
Zn-BDC MOF Cu-BDC MOF	Mechanical Mechanical	-	Naphthalene Anthracene Naphthalene Anthracene	100	88 50 84 52	87 52 84 52	210 min 120 min 210 min 120 min	3	[211]
UiO-66(Zr) NH_2_-UiO-66(Zr)	Solvothermal	1420 985	Anthracene Chrysene Anthracene Chrysene	4	99 96 98 96	24 22 24 19	25 min 25 min 30 min 30 min	5 5	[187]
MIL-88(Fe) NH_2_-MIL-88(Fe)	Microwave	1240 941	Pyrene Pyrene	4	99 96	24 23	40 min	5	[212]
MIL-88(Fe) NH_2_-MIL-88(Fe)	Microwave	1240 941	Chrysene Chrysene	4	99 95	24 22	25 min	5	[188]
MIL-88(Fe) NH_2_-MIL-88(Fe) Mixed-MIL-88(Fe)	Microwave	1240 941 1025	Anthracene Anthracene Anthracene	4	98 92 96	24 21 23	25 min	-	[189]
			**PFCAs**						
ZIF-7 ZIF-8 ZIF-L	Room temperature	14 1291 12	Perfluorooctanoic acid	250	40 45 97	26 214 295	60 min	-	[213]
Basolite A-100	Commercial	630	Perfluorooctanoic acid	1	100	169		4	

**Table 4:** MOFs and composites reported for the photocatalytic degradations of phenols, herbicides, pesticides, and other miscellaneous organic pollutants.

MOF	Synthesis Method	Surface Area (m^2^ g^−1^)	Bandgap (eV)	Pollutants	Concentration (mg L^−1^)	Light Source	(%) Removal	Irradiation Time	Reused	Ref
				**Phenolics**						
NH_2_-MIL-125 (Ti)@Bi_2_M	Solvothermal	88	1.89	Dichlorophen	10	Visible	93	180 min	-	[214]
[CoNi(m_3_-tp)_2_(m_2_-pyz)_2_] MOF/CuWO_4_	Hydrothermal	1054 801	2.5 2.4	4-nitrophenol	10	Visible	24 81	105 min	6	[152]
MIL-88B(Fe) CNT@MIL-88B(Fe)	Hydrothermal Hydrothermal	118	-	Phenol	25		55 100	30 min 10 min	3	[215]
CdS@NH2-MIL-125(Ti)	Solvothermal	1375	2.36	Phenol	180	Visible	-	120 min	5	[147]
HOQ@MOF-5(Zn)	Room temperature	-	3.12	Phenol	1	Visible	100	70 min	5	[216]
MIL-100(Fe)@ZnO	Solvothermal	654	2.63	Phenol, Bisphenol A	5	Visible	95 84	120 min	5	[191]
MIL-101-NH_2_@TpMA UiO-66-NH_2_@TpMA	Hydrothermal Hydrothermal	129 531	2.12 2.01	Bisphenol A	50	Visible	99 82	240 min 240 min	5 5	[192]
MIL-88(Fe)/PS/UV	Microwave	-	1.78	Bisphenol A	10	Visible	100	30 min	3	[217]
MIL-101(Fe) Pd@MIL-100(Fe)	Hydrothermal	2006 2102	-	Bisphenol A	20	Visible	47 68	240 min	4	[218]
Cu-hemin-MOFs/BN	Room temperature	-	-	Bisphenol A	40	Visible	99	30 min	4	[219]
laccase@HKUST-1(Cu)	Immobilization	-	-	Bisphenol A	200	Visible	100	4 h	10	[203]
AQS-NH-MIL-101(Fe)	Solvothermal	-	-	Bisphenol A	60	Visible	98	180 min	3	[220]
				**Pesticides**						
UiO- 66@WG	Solvothermal	380	2.3	Malathion	20	Visible	83	70 min	4	[221]
AgIO_3_/MIL-53(Fe)	Room temperature	208	2.43	Malathion Chlorpyrifos	20	Solar	93 98	120 min	-	[222]
Fe_3_O_4_@MOF-2	Room temperature	-	-	Diazinon	30	Visible	99	60 min	15	[223]
MIL-53(Fe)	Solvothermal	668	2.89	Thiamethoxam	5	Visible	96	60 min	-	[190]
HKUST-1(Cu) ZrO_2_@HKUST-1(Cu)	Room temperature Solvothermal	1484 1152	3.87 2.27	Cyhalothrin	60	Visible	34 100	6 h	4	[208]
				**Herbicides**						
MIL-100(Fe)@ZnO	Solvothermal	654	2.63	Atrazine	5	Visible	79	120 min	5	[191]
TiO_2_@NH_2_-MIL-101(Cr)	Solvothermal	-	-	Atrazine	30	Visible	45	60 min	-	[84]

It has long been recognized that the catalytic activity of enzymes can be extended by immobilizing onto solid supports, such as polymers and inorganic materials. The superior performance of MOF HKUST-1 for the encapsulation of the enzyme laccase to enhance its catalytic activity, stability, and reusability compared with other conventional polymers or inorganic carriers was demonstrated by Zhang et al. (2020). The MOF not only acted as protective layer against high temperatures, continuous operation, and long-term storage but also could enhance the accessibility of active site of laccase due to its flower-like structure and high exposed surface area. The laccase@HKUST-1 still maintained 75.9% of its original degradation efficiency after 10 cycles, suggesting the effectiveness of the MOF to act as a protective layer to protect the laccase against the possible industrial environment. Unfortunately, the rapid breakdown of bisphenol using this composite material did not materialize (4 h).

### 3.3. MOFs and Composites for Adsorption and Photocatalytic Degradation of Pharmaceutical and Personal Care Products (PPCPs)

PPCPs are produced and used worldwide primarily for the remediation of ailments, as supplements, and as body care. These chemicals are usually discharged as wastewater from the manufacturing industries, hospitals, landfill leachates into the environment, either in their native form or as metabolites. The fundamental pathway for the release of these contaminants is through excretions. Thus, municipal wastewater is the major route bringing human pharmaceuticals into the environment. Of the various class of pharmaceuticals, antibiotics, such as penicillin, amoxicillin, tetracyclines, sulfonamides, etc., are found to be persistent in water due to their resistance to biological treatments from wastewater treatments plants. They usually remained untreated in the municipal wastewater for a long time; hence, they pose toxic effects even at low concentrations (ng L^−1^). Although the concentration of these pharmaceutical residues in the environment is low, its uninterrupted input to the environment may result in the long-term risk for terrestrial and aquatic organisms. In human beings, these pollutants may cause mutations in the genomic texture by disrupting the endocrine glands; hence, they are classified as endocrine disruptors.

**Figure 9:**
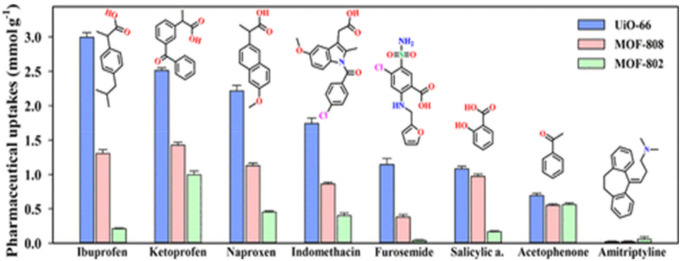
Adsorption capacities of UiO-66(Zr), MOF-808(Fe), and MOF-802(Fe) for the removal of pharmaceutical drugs from water. Reproduced with permission from Reference [224].

The applications of MOFs as adsorbents, as well as photocatalysts, for the remediation of PPCPs have been reported (Table 5). Many MOFs were proven to be efficient for the adsorption of these pollutants within short time with high removal capacities. Similarly, the use of pristine MOFs and their corresponding functionalized derivatives and composites have been studied. MOFs composites have demonstrated better photocatalytic activities than the pristine MOFs. Some of these MOFs have also displayed good reusability which could be employed for industrial and large-scale applications. Figure 9 illustrates the versatility of MOFs, such as UiO-66(Zr), MOF-88(Fe), and MOF-808(Fe), for the removal of some common pharmaceuticals [224].

**Figure 10:**
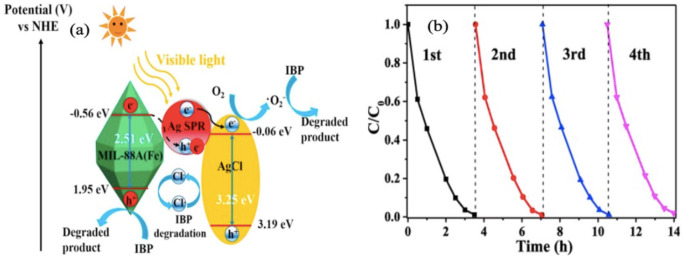
(**a**) Mechanism for photocatalytic degradation of ibuprofen using MIL-88(Fe) and Ag/AgCl@MIL-88(Fe) and (**b**) the reusability of the composites. Reproduced with permission from Reference [251].

Photocatalysts of high porosity, ordered crystallinity, visible light harvesting capabilities and mechanical stability are desirable for the complete mineralization of the pharmaceutical drugs. The presence of the metallic node and organic linker can enhance the utilization of the solar energy through HOMO and LUMO interactions. The interactions generate the photon energy that are responsible for the excites the electrons from the contaminants to produce the active species of H^+^ and OH^−^ that mineralize the organic species. Figure 10 illustrates the mechanism for the photocatalytic degradation of ibuprofen using MIL-88(Fe) and corresponding composites, Ag/AgCl@MIL-88(Fe). The incorporation of AgCl into the framework or the MIL-88(Fe) MOF caused reduction in the bandgap (2.51 eV) of the MOF, which improved the photocatalytic capability of the MOF [251]. The applications of MOFs and their composites for the photocatalytic degradation of pharmaceutical drugs is highlighted in Table 6. In most cases, several hours are required for the complete mineralization of the pharmaceuticals.

## 4. Patent Search

**Figure 11:**
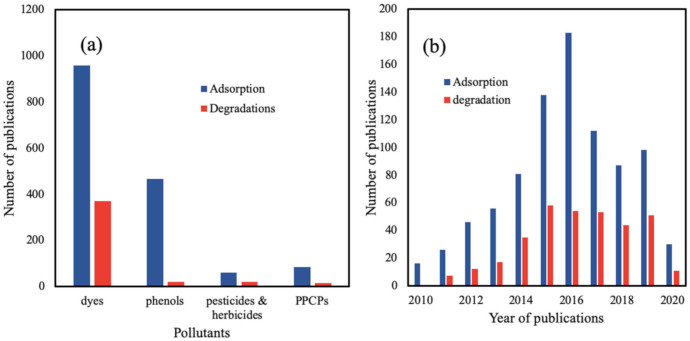
Patents granted from 2010 to 2020 on the adsorption and photocatalytic degradation using MOFs-based materials of (**a**) some emerging pollutants and (**b**) dyes. Data obtained from the lens.org using keywords MOFs, adsorption, photocatalytic degradation, dyes, phenols, PPCPs, pesticides, and herbicides.

The diversity in MOFs and their versatile functionalities has prompted researchers to explore their potentialities in synthesis and applications. Thus, number of literatures has been written and patented on the synthesis and applications of MOFs and their composites. The advancement in the synthesis and characterizations of MOFs and frontier applications in adsorption and photocatalytic degradation. The area of research remains active among community of scientists and engineers. Thus, the number of published articles for MOFs application in wastewater remediations have been well patented. A search using the website lens.org reveals that most patents were granted for the past 10 years on adsorption using MOFs were on dyes, followed by phenols, PPCPs, and then pesticides and herbicide. Similarly, with the photocatalytic degradation (Figure 11a). Patents granted for the adsorption and photocatalytic degradation of dyes using MOFs-based materials are shown in Figure 11b. The growth was exponential until 2016, with a gradual decrease from then on. The reason for the decreased in patenting could be due to the discovery of a large number of promising MOFs for various laboratory and pilot-scale wastewater applications.

## 5. Conclusions

The motivation for the development of improved technologies for the remediation of waters is driven by the frequent occurrence of emerging pollutants in drinking water. This is because the conventional wastewater treatment facilities are ill-equipped for the complete removal of these pollutants in water. Adsorption using conventional adsorbents, despite being the gold standard in water treatment technology, is not suited for the task. MOFs and/or their composites, on the other hand, have shown very encouraging results not only as super adsorbents but also as super photocatalysts. The extreme porosity and large interior surface area of MOFs offer unique prospects for adsorption and photocatalysis. Unlike conventional adsorbents which rely to a large extent on the unspecific van der Waals force, the simultaneous use of various interactions, such as cationic, π–π stacking, hydrogen-bonding, and Van der Waals interactions, has been associated with MOFs adsorption. MOFs can also offer more selectivity to the organic pollutants than other conventional adsorbents due to the orientation of their frameworks. They provide large number of pores with uniform sizes. The ‘breathing effects’ of MOFs cavity allow for the adsorption of larger molecules of pollutant from wastewater. For photocatalytic application, their visible light adsorption capacity and moderate bandgap has been commended. To a larger extent, composites of MOFs offer great advantage than their pristine forms due their multiple functionalities. Thus, MOFs have proven to be promising materials for adsorption and photocatalytic degradation of different classes of organic pollutants.

A few start-up companies which are predominantly spin-offs from university laboratories and the German chemical company BASF have started commercializing several kinds of MOFs, mainly for applications as gas storage and adsorption of toxic gases. MOFs, such as MOF-5, MIL-53, HKUST-1, ZIF-90, and UIO-66, can be obtained from the open market. It must be pointed out that most evaluations cited in this article were conducted under normal laboratory conditions. The actual performance of the MOFs in real water samples with complex matrices, such as wastewater and under industrial-scale operations, are virtually unknown. For commercial exploitation, it would perhaps be easier for these adsorbents materials to be applied as super filters in the household water purification system due to the smaller amounts of adsorbents/photocatalysts required. For large-scale productions, such as wastewater treatment facilities, the cost will be a primary factor on the commercial exploitation of these materials. However, if savings from mass production and reusability are factored, it might be cost-effective on the long run. The use of cheaper metals (e.g., potassium, sodium) and, at the same time, not compromising the qualities of the MOFs will be the way forward. Photocatalysts can able to harness direct sunlight and significantly reduce the degradation time are much welcome. Other major challenges that must be overcome are the often complicated and lengthy synthesis processes, poor long-term physicochemical stability of the MOFs, and the limited prospects for reuse. Typical of any new materials, long term safety issues, such as the liberation of chemicals and metals from the degradation of MOFs, as well as risks to exposure to trapped organic solvents (e.g., chloroform, acetone, dimethylformamide), are virtually unknown.

The application of MOFs for industrial wastewater treatments have been established. The major form for the adsorbents and photocatalysts desired includes pellets, spherical, mold, nanorods, beads, etc. Thus, the use of MOFs composites has demonstrated many advantages, particularly in photocatalysis, where low bandgap is required. The requirements include high surface area and small pore diameters with distinct pore structures to enable faster transport of the MOFs in the aqueous phase. Along with that, thermal stability, abrasion, and moisture resistance are prerequisites to the industrial application of the MOFs.

Thus, adsorption with simultaneous photocatalytic degradation under sunlight irradiation is certainly a novel idea as it offers a complete solution to the problem of removal of pollutants from wastewater and their safe remediation into environmentally benign species. MOFs and their composites seem destined to play these roles.

## Figures and Tables

**Table 1:** MOFs reported for the adsorption of dyes.

Type of MOF	Synthesis Method	Surface Area (m^2^ g^−1^)	Pollutants	Concentration (mg L^−1^)	% Removal	Qe (mg g^−1^)	Equilibrium Time	Reused	Ref
Fe-BTC	Solvothermal	877	Orange II	50	92	207	80 min	4	[52]
MIL-53(Fe)	Solvothermal	53	Methyl orange	100	77	77	60 min	3	[100]
MOF-235(Fe)	Solvothermal	-	Methyl orange Methylene blue	30	-	477 187	250 min	-	[101]
MIL-125(Ti)	Solvothermal	1108	Crystal violet	40	-	130	180 min	-	[102]
MIL-101(Cr)	Hydrothermal	3514	Methylene blue Methyl red	30 300	-	11 247	30 min 30 min	- -	[103]
MIL-101(Cr)	Microwave	2410	Reactive yellow Reactive black Reactive red Reactive blue	300	100	386 377 390 397	24 h	-	[104]
MIL-100(Fe) MIL-100(Cr)	Hydrothermal Hydrothermal	17701760	Methyl orange Methylene blue Methyl orange Methylene blue	30 30	85 100 8 100	1045 736 212 645	3 days 22 days	- -	[99]
MIL-101(Cr) MIL-101(Cr)-SO_3_H	Hydrothermal Hydrothermal	3016 1546	Fluorescein sodium Safranine Fluorescein sodium Safranine	100 100	- - - -	280 701 114 425	700 min 700 min 700 min 700 min	4 4	[105] [105]
Cu-BTC	Hydrothermal	521	Methylene blue	200	-	96	40 min	4	[106]
Cu-BTC MOF Cu-BTC@GO Cu-BTC@CNT Fe_3_O_4_/Cu-BTC@GO	Solvothermal	856508123176	Methylene blue	100	- - - -	67 152 172 136	12 h	-	[107]
Ce(III)-doped UiO-67	Solvothermal	1911	Methylene blue Congo red Methyl orange	100	95 96	399 800 401	80 min	4 4	[98]
AlF-MOF AlF-GO AlF-rGO	Hydrothermal	973 918 952	Congo red	50	99	93 102 179	30 min	-	[108]
NH2 -MIL-125(Ti)	Solvothermal	1350	Basic blue Methylene blue Basic red	20	93 97 99	1257 862 1296	30 min	3	[109]
NH_2_-UiO-66(Zr)	Solvothermal	954	Methylene blue	200	88	321	15 min	6	[30]
UiO-66(Zr)	Solvothermal	1244	Rhodamine Blue	20	91	90	200 min	5	[110]
Zn-MOF	Room temp	1046	Methylene blue	10	98	326	60 min	4	[111]
CPM-97(Fe)	Solvothermal	1397	Congo red	40	100	831	30 min	3	[112]
MIL-53(Fe)	Solvothermal	23	Methyl red	100	78	76	60 min	3	[100]
MIL-101(Cr)	Hydrothermal	2664	Xylenol orange	400	90	159	30 min	3	[113]
BTB-Mn	Solvothermal	3143	Methylene blue	15	89	308	120 min	6	[114]
NOTT-102(Cu)	Solvothermal	3006	Methylene blue	20	97	850	24 h	3	[115]
Ni-Zn-MOF	Solvothermal	57	Congo red	30	-	461	300 min	5	[116]
Cu-MOF/Fe_3_O_4_	Solvothermal	34	Malachite green	50	90	114	60 min	5	[117]
Ni-MOF/GO	Ball milling	70	Congo red	200	-	2489	300 min	-	[118]
PEI-modified Cu-BTC	Hydrothermal	785	Congo red Acid blue	1200 100	100 100	2578 132	200 min	6 6	[78]
PED-MIL-101(Cr) PED-MIL-101(Cr)	Hydrothermal	3491 3296	Methyl orange Methyl orange	50 50	NA NA	160 194	250 min 250 min	3 3	[119]
Ac-HKUST-1	Solvothermal	-	Crystal violet Disulfine blue Quinoline yellow	10 10 10	100 91	133 130 65	4 min	-	[120]
MIL-101(Fe)@PDopa@Fe_3_O_4_	Solvothermal	-	Methyl red Malachite green	100 100	92 100	833 1250	30 min 60 min	4 4	[121]
H_6_P_2_W_18_O_62_ /MOF-5	Hydrothermal	395	Methylene blue	20	97	52	10 min	-	[122]
Fe_3_O_4_@MIL-100(Fe)	Solvothermal	730	Methylene blue	20	83	221	24 h	4	[123]
NENU/GO	Solvothermal	380	Basic red 46	5	88	130	6 min	-	[124]
MIL-68(Al)/PVDF	Casting	-	Methylene blue	10	96	61	360 min	6	[125]
NH_2_-UiO-66(Zr)	Solvothermal	247	Safranin	135	100	39	480 min	4	[126]
MIL-101(Cr) TiO_2_-MIL-101(Cr)	Hydrothermal	2361 531	Methylene blue	20	-	9 21	50 min	-	[127]

**Table 2:** MOFs and MOF composites for the photocatalytic degradation of dyes.

MOF	Synthesis Method	Surface Area (m^2^ g^−1^)	Bandgap (eV)	Pollutants	Concentration (mg L^−1^)	Light Source	(%) Removal	Irradiation Time	Reused	Ref
MIL-88(Fe)	Hydrothermal	-	2.05	Methylene blue	32	Visible	-	50 min	4	[132]
NH_2-_MIL-88(Fe)	Microwave	164	-	Methylene blue	20	Visible	98	60 min	5	[50]
MIL-100(Fe)	Hydrothermal	5	-	Basic blue	15	Ultraviolet	99	180 min	3	[133]
MIL-125(Ti)	Microwave	-	3.14	Methylene blue	-	Visible	97	360 min	-	[134]
MIL-101(Fe) MIL-100(Fe) MIL-53(Fe) MIL-88B(Fe)	Solvothermal Solvothermal Solvothermal Solvothermal	2986 1798 965 19	- - - -	Acid orange	80	Visible	95 88 62 23	120 min	3	[135]
MIL-53(Fe) Ni-MIL-53(Fe)	Solvothermal	300 480	2.59 2.24	Rhodamine blue	14.4	Visible	81 91	180 min	-	[136]
MIL-101(Cr) TiO_2_-MIL-101(Cr)	Hydrothermal	2361 531	2.3 2.59	Methylene blue	20	Ultraviolet	43 100	30 min	-	[127]
NH_2_-MIL-88B(Fe)	Microwave	164	-	Methylene blue	20	Visible	98	45 min	5	[50]
NT/MIL-100(Fe)	Hydrothermal	1414	- -	Methylene blue Rhodamine blue	-	Visible	99 94	180 min	4	[137]
PCN/MIL-100(Fe)	Hydrothermal	1252	-	Methylene blue Rhodamine blue	10	Visible	75 80	200 min	-	[131]
TiO_2_@MIL-101(Fe)	Hydrothermal	1919	-	Methyl orange	150	Ultraviolet	99	50 min	-	
NH_2_-MIL-125(Ti) CQDs/NH_2_-MIL-125(Ti)	Hydrothermal	487 198	2.43 2.33	Rhodamine blue	10 10	Visible	64 100	120 min 120 min	7 7	[90]
NH_2_-MIL-53(Al) NH_2_-MIL-53(Al)/ RGO/PS	Hydrothermal	1051 95	2.7 2.4	Methylene blue	30	Visible	41 59	210 min	3	[138]
MIL-100(Fe)@Bi_2_S_3_	Microwave	702	1.75	Rhodamine blue	10	Visible	98	60 min	4	[139]
MOF-199	Solvothermal	343	5.43	Basic blue	20	Ultraviolet	-	180 min	-	[140]
MOF-199 MOF-199-NH_2_/BaWO_4_	Hydrothermal	- -	3.2 3	Methyl orange	10 10	Ultraviolet	38 98	50 min	-	[141]
MOF-1	Solvothermal	-	3.0	Methyl violet	10	Ultraviolet	74	100 min	-	[142]
HU11(Pr)	Solvothermal	-	3.3	Crystal blue	220	Visible	100	24 h	-	[143]
UiO-66/g-C_3_N_4_	Mechanical	384	2.72	Methylene blue	10	Visible	-	180 min	6	[144]
Bi_2_MoO_6_/UiO-66(Zr)	Hydrothermal	726	2.45	Rhodamine blue	10	Visible	96	120 min	3	[145]
In_2_S_3_/UiO-66(Zr)	Solvothermal	802	1.4	Methyl orange	15	Visible	96	40 min	5	[146]
CdS@NH_2_-MIL-125(Ti)	Solvothermal	1247	2.36	Rhodamine blue	180	Visible	97	120 min	-	[147]
Ag_3_VO_4_/Cu-MOF/GO	Room temperature	6	-	Acid blue	10	Visible	100	120 min	3	[148]
BiVO_4_/Fe-MOF/GO	Microwave	33	2.18	Rhodamine blue	15	Visible	-	60 min	4	[1]
AgBr@HPU-4	Room temperature	-	-	Methylene blue Methyl orange	12.75 12.75	Visible	95 92	60 min 120 min	5 5	[149]
BiVO_4_/MIL-53(Fe)	Solvothermal	33	2.18	Rhodamine blue	15		-	60 min	4	[1]
Ag_3_PO_4_/AgBr/Ag-HKUST-1	Solvothermal	1		Methylene blue Acid orange Eosin red	15	Visible	92 90 90	80 min	3	[150]
Ag_3_PO_4_/Bi_2_S_3_-HKUST-1	Solvothermal	-	2.07	Trypan blue vesuvine	25	Visible	98 99	25 min	-	[151]
MOF/CuWO_4_	Hydrothermal	801	2.4	Methylene blue	10	Visible	98	135 min	6	[152]
QD/Eu-MOF	Room temperature	-	2.29	Rhodamine blue	2	Ultraviolet	90	50 min	-	[153]
Resin/FeBTC	Hydrothermal	-	2.31	Rhodamine blue Methylene blue	400	Visible	99 67	30 min	5	[154]
MIL-53(Fe)	Solvothermal	-	2.43	Rhodamine blue	1580	Visible	85	120 min	5	[155]
MIL-53(Fe)	Solvothermal	-	3.87	Methylene blue	128	Visible	99	20 min	5	[156]
MIL-53(Fe)	Solvothermal	38	2.69	Rhodamine blue	10	Visible	-	180 min	-	[157]
MIL-53(Fe)	Solvothermal	89	-	Orange green	0.2	Visible	98	90 min	5	[158]
MIL-100(Fe)@MIL-53(Fe)	Sonochemical	315	1.84	Methyl orange	10	Visible	98	180 min	5	[159]
[CoNi(m3-tp)_2_ (m_2_-pyz)_2_] MOF/CuWO_4_	Hydrothermal	1054 801	2.5 2.4	Methylene blue	10	Visible	32 98	135 min	6	[152]
UiO-66(Zr) α-Fe_2_O_3_@UiO-66(Zr)	Solvothermal	1487 1204	-	Methylene blue	128	Visible	-	50 min	3	[160]
UiO-66(Zr) CuS/UiO-66(Zr)	Solvothermal	- -	3.5 2.01	Rhodamine blue	10	Visible	50 90	60 min	3	[161]
NiFe_2_O_4_/MIL-53(Fe)	Solvothermal	43	-	Rhodamine blue	4.7	Visible	95	180 min	-	[162]
MIL-88(Fe) TiO_2_NS@MIL-100(Fe)	Hydrothermal	1670 725	2.6 2.87	Methylene blue	50	Visible	-	60 min	4	[163]

**Table 5:** Adsorptions of PPCPs onto MOFs and their composites.

Type of MOF	Synthesis Method	Surface Area (m^2^ g^−1^)	Pollutants	Concentration (mg L^−1^)	% Removal	Qe (mg g^−1^)	Equilibrium Time	Reused	Ref
A100(Al) MOF	Commercial	630	Carbamazepine Ibuprofen	2 2	95 75	65 50	2 h 2 h	4	[225]
NH_2_-MIL-68(In)	Hydrothermal	655	p-arsanilic acid	20	77	78	4 h	4	[226]
Fe_3_O_4_@MIL-100(Fe)	Microwave	1245	Diclofenac	100		248	4 h	-	[227]
MIL-101 (Cr) ED-MIL-101(Cr) AMSA-MIL-101(Cr)	Hydrothermal	3014 2322 2255	Naproxen Clofibric Naproxen Clofibric Naproxen Clofibric	13 100	-	131 315 93 105 154 347	2 h	4	[228]
PCN-134(Zr)	Solvothermal	756	Diclofenac	30	-	-	20 min	-	[229]
[Cu(BTTA)]n.2DMF	Solvothermal		Diclofenac Chlorpromazine Amodiaquine	1200 1000 1000	- - -	650 67 72	7.5 h 5 h 5 h	3	[230]
[Zn_2_(fum)_2_(bpy)] [Zn4O(bdc)_3_]	Mechanical Solvothermal	-	Amodiaquine	25	-	0.5 48	3 h	-	[231]
[Zn_6_(IDC)_4_(OH)_2_(Hprz)_2_]n	Hydrothermal	889	Ampicillin Amoxicillin Cloxacillin	60	93 88 89	-	4 h	4	[232]
PCN-222(Zr)	Solvothermal	2917	Chloramphenicol	500	99	370	58 sec	-	[233]
PCN-128Y(Zr)	Solvothermal		Tetracycline	44	56	400	30 min	-	[234]
MIL-53(Al)	Hydrothermal	1401	Dimetridazole	40	90	467	10 min	5	[3]
MOF-5	Room temperature	2510	Tetracycline	50	97	233	45 min	-	[235]
MIL-53(Cr) MIL-53(Al)	Solvothermal	500 500	Sulfonamide	20	99 98	0.4 0.4	1 h	3 3	[236]
MIL-53(Fe)/Fe3O4.	Solvothermal	76	Doxycycline	300	100	320	30 min	5	[237]
MIL-101(Cr) MIL-53(Cr)	Hydrothermal Hydrothermal	2810 398	Clofibric acid Carbamazepine Clofibric acid Carbamazepine	20	-	144 35 137 31	1 h	-	[238]
MIL-101(Fe) MIL-100(Fe) MIL-53(Fe)	Hydrothermal Hydrothermal Solvothermal	253 1203 21	Tetracycline	50	55.1 44 11	52 43 12	40 min	4	[239]
Ni-MIL-53(Fe)	Solvothermal	-	Doxycycline	150	88	684	12 h	5	[240]
MIL-101(Cr) Urea-MIL-101(Cr)	Hydrothermal	3030 1970	Dimetridazole	10	-	141 185	4 h	4	[241]
Pd@MIL-100(Fe)	Hydrothermal	2102							
MWCNT/NH_2_-MIL-53(Fe)	Solvothermal	126	Tetracycline Chlortetracycline	20	- -	368 254	12 h	4	[242]
MWCNT/MIL-53(Fe)	Solvothermal	60	Tetracycline Oxytetracycline Chlortetracycline	20	-	364 326 181	10 h	4	[243]
UiO-66(Zr) NH_2-_UiO-66(Zr)	Solvothermal	1171 646	Ibuprofen Naproxen Ibuprofen naproxen	9	- -	127 89 51 40	4 h 4 h	-	[244]
UiO-66(Zr) In_2_S_3_/UiO-66(Zr)	Solvothermal	389 75	Tetracycline	40	-	51 61	1 h	3	[245]
UiO-66(Zr)	Solvothermal	1155	Sulfonamide	100	-	417	10 min	4	[246]
Fe_3_O_4_/HKUST-1(Cu)	Solvothermal	328	CiprofloxacinNorfloxacin	20	98 99	538 513	30 min	10	[46]
Zn(TDC)(4- BPMH)]n·n(H_2_O)	Sonochemical	235	Dichlorophenol Amoxicillin	50	99 99	- -	3 h	- -	[196]
Ni/Co-MOF@CMC	Microwave	-	Tetracycline	30	80	625	5 min	-	[247]
MIL-68(Al)/GO	Hydrothermal	1267	Tetracycline	50	-	173	6 h	3	[248]
MIL-101(Cr) GnO/MIL-101(Cr)	Hydrothermal	- 3308	Naproxen Ketoprofen Naproxen Ketoprofen	50	-	112 80 171 140	12 h	4	[249]
Cu-DTO	Room temperature	120	Tartrazine	200	98	255	40 min	7	[250]

**Table 6:** MOFs and composites employed for photocatalytic degradations of pharmaceutical drugs from wastewater.

MOF	Synthesis Method	Surface Area (m^2^ g^−1^)	Bandgap (eV)	Pollutants	Concentration (mg L^−1^)	Light Source	(%) Removal	Irradiation Time	Reused	Ref
MIL-53(Fe)	Solvothermal	1890	2.75	Tetracycline	10	Visible	97	2 h	4	[252]
MIL-101(Fe) MIL-100(Fe) MIL-53(Fe)	Hydrothermal Hydrothermal Solvothermal	253 1203 21	1.88 2.06 1.97	Tetracycline	50	Visible	97 57 41	3 h		[239]
MIL-100(Fe)@Fe_3_O_4_ MIL-100(Fe)@Fe_3_O_4_/CA	Hydrothermal	725 389	2.49 1.76	Tetracycline	10	Visible	42 85	3 h	7	[253]
MIL-88(Fe) Ag/AgCl@MIL-88(Fe)	Solvothermal	26 139	2.51 2.23	Ibuprofen	10	Visible	45 93	3.5 h	4	[251]
CdS@NH_2_-MIL-125(Ti)	Solvothermal	1375	2.36	Oxytetracycline	180	Visible	-	2 h	5	[147]
MIL-101(Fe) Pd@MIL-100(Fe)	Hydrothermal	2006 2102	-	Theophylline Ibuprofen Theophylline Ibuprofen	20	Visible	88 92 100 100	2.5 h	4	[218]
UiO-66(Zr) In_2_S_3_/UiO-66(Zr)	Solvothermal	389 75	3.70 1.92	Tetracycline	40	Visible	56 79	1 h	3	[245]
In_2_S_3_/UiO-66(Zr)	Solvothermal	48	2.2	Tetracycline	30	Visible	85	1 h	5	[146]
MIL-100(Fe) Fe_3_O_4_@MIL-100(Fe)	Hydrothermal Microwave	1766 1245	- -	Diclofenac	60	visible	100 99		- -	[227]
Vis/MIL-53(Fe)/Fe(III)/SPC	Solvothermal	-	2.91	Sulfamethazine	0.2	Visible	90	1 h	-	[254]
1T- MoS_2_@MIL-53(Fe)	Solvothermal	337	0.7	Ibuprofen	10	Visible	100	2 h	5	[255]
MIL-68(In)-NH_2_ g-C_3_N_4_/MIL-68(In)-NH_2_	Solvothermal	659 537	2.81 2.65	Ibuprofen	20	Visible	93 68	2 h	-	[248]
MIL-125ML MIL-125ML/gCN	Solvothermal	1001 725	2.86 2.68	Cefixime	20	Visible	48 74	2 h	4	[256]
UiO-66-NH_2_ CNT/N-TiO_2_/UiO-66-NH_2_	Hydrothermal	708 288	2.17	Ketoprofen	50	Visible	41 96	2 h	-	[257]
MIL-101(Cr) α-Fe_2_O_3_/MIL-101(Cr)	Hydrothermal	2518 949	3.25 3.62	Carbamazepine	30	Visible	- -	3 h	4	[258]
MIL-53(Fe)	Solvothermal	184	-	Clofibric acid Carbamazepine	40	Visible	98 90	4 h	4	[259]

## Abbreviations

BaWO_4_	Barium tungstate
BET	Brunner Emmett Teller
COF	Covalent organic framework
CNTs	Carbon nanotubes
CPs	Coordination polymers
CQDs	Carbon quantum dots
EDCs	Endocrine disrupting compounds
GO	Graphene oxide
HKUST	Hongkong University of Science and Technology
HOMO	Highest occupied molecular orbital
HPLC	High performance liquid chromatography
LMCT	ligand to metal cluster charge transition
LUMO	Lowest occupied molecular orbital
MIL	Material institute Lavoisier
MIPs	Molecularly impregnated polymers
MNPs	Metal-oxide nanoparticles
MOFs	Metal-organic frameworks
PAHs	Polycyclic aromatic hydrocarbons
PDI	Pyromellitic diimide
PFAS	Perfluoroalkyl substances
PFCs	Perfluorinated compounds
PFCAs	Perfluoro carboxylic acids
PFOA	Perfluorooctanoic acid
PFOS	Perfluorooctane sulfonates
PPCPs	Pharmaceutical and Personal Care Products
RGO	Reduced graphene oxide
UiO	Universiti i Oslo
USEPA	United-states environmental protection agency
ZIFs	Zeolite imidazole framework
